# Patient satisfaction with radio-frequency identification (RFID) tag localization compared with wire localization for nonpalpable breast lesions: the RFID trial

**DOI:** 10.1186/s12885-025-13453-0

**Published:** 2025-01-22

**Authors:** Raphaël Pete, Céleste Pinard, Fanny Sirodot, Ioana Molnar, Margot Dressaire, Angeline Ginzac, Catherine Abrial, Xavier Durando, Marielle Tekath

**Affiliations:** 1 Unité de Sénologie, Centre Jean PERRIN, Clermont-Ferrand, France; 2 Service de Radiologie, Centre Hospitalier Universitaire, Clermont-Ferrand, France; 3 Division de Recherche Clinique, Délégation Recherche Clinique & Innovation, Centre Jean PERRIN Division de Recherche Clinique, 58, rue Montalembert, Clermont-Ferrand, 63011 France; 4 Université Clermont Auvergne, INSERM UMR 1240 « Imagerie Moléculaire et Stratégies Théranostiques », Centre Jean PERRIN, Clermont-Ferrand, France; 5 Centre d’Investigation Clinique, UMR501, Clermont-Ferrand, France; 6 Service de chirurgie oncologique, Centre Jean PERRIN, Clermont-Ferrand, France; 7 Département d’oncologie médicale, Centre Jean PERRIN, Clermont-Ferrand, France

**Keywords:** Breast cancer, Non-palpable breast lesions, Radio-frequency tag, Wire-guided-localization, Breast conservation surgery

## Abstract

**Background:**

Most breast cancers are detected at an early stage in which case conservative surgery is indicated. An accurate preoperative localization technique is essential for conservative surgery of non-palpable breast lesions. Currently, the gold standard technique is wire localization (WL). However, this technique has well-known drawbacks. Several wire-free techniques have been developed to overcome these drawbacks; one technique is localisation by Radiofrequency Identification (RFID). The purpose of this clinical trial was to assess the superiority of RFID tags (HOLOGIC) in terms of patient satisfaction, over wire localization of non-palpable breast lesions.

**Methods:**

This was a single-centre, prospective, controlled and non-interventional trial. Patients were followed from their inclusion at the time of the preoperative consultation to the postoperative consultation, one month after surgery. Data on anxiety and satisfaction was collected from patients and clinicians using questionnaires, and clinical data was collected from the medical files. The primary outcome was the patients’ satisfaction scores, assessed using a visual analogue scale.

**Results:**

Eighty patients were sequentially enrolled in two groups: the wire group (*n* = 40) and the RFID group (*n* = 40). One patient from the RFID group was excluded from the analysis because of a substantial migration during deployment. On a 10-point Visual Analogue Scale, the patients’ median satisfaction score was 9.8 (IQR = 1.32) for the wire group and 10 (IQR = 0.07) for the RFID group (*p* < 0.001). A reduction in pain between device insertion and surgery was observed in the RFID group (*p* = 0.009). The median placement time was shorter in the RFID group (15 min, IQR = 6) than in the wire group (20 min, IQR = 30) (*p* = 0.01).

**Conclusion:**

Our results show a statistically significant difference in median patient satisfaction score with the localization of non-palpable breast cancer lesions using RFID tags compared to the use of the WL. Although our results did not show clinically significant outcomes in terms of satisfaction, RFID tags are a reliable alternative to WL and simplify the organization of patients’ healthcare trajectories.

**Trial registration:**

ClinicalTrials.gov ID; NCT04750889 registered on February 11, 2021. https://clinicaltrials.gov/ct2/show/NCT04750889?term=rfid&draw=2&rank=1

**Supplementary Information:**

The online version contains supplementary material available at 10.1186/s12885-025-13453-0.

## Background

Most breast cancers are detected at an early stage, primarily among young patients, in which case conservative surgery is indicated. In the case of a lesion that cannot be palpated, the radiologist needs to locate the lesion prior to surgery. In France and worldwide, wire localization remains the gold standard technique for surgical guidance for non-palpable breast lesions in conservative breast surgery. However, it has several well-known drawbacks, including stringent time requirements for surgeons and radiologists. To avoid any risk of infection or wire displacement when pulling the thread out of the patient’s skin, the surgery must be carried out on the same day the device is inserted, or on the following day. This requires considerable coordination between the radiologist and the surgical teams, which is not always easy to achieve. It is also well known that this device can cause a certain amount of anxiety among patients [1]. Consequently, this has led to the development of wire-free alternatives, such as Radiofrequency Identification (RFID), radioactive seed, radar reflector and magnetic localization [2].

In this study, we focused on RFID localization. RFID tags appear as a promising alternative since they could improve patient comfort and reduce the scheduling constraints imposed by wire localization (WL). Most of the existing publications on this new device have focused exclusively on data relating to the performance and efficacy of the tag without directly comparing it to WL. Most of the data collected retrospectively relates to the success of deployment, the identification and extraction of the tag, the state of the surgical margins and the occurrence of re-excision in cohorts of patients presenting non-palpable breast lesions [3–5]. Few studies have been carried out to compare this data in two different cohorts: tag vs. wire guidance. Only two American retrospective studies have compared the safety RFID localization to that of WL [6, 7] and one recent prospective study compared RFID localization with a magnetic localization technique (Magseed^®^) and WL, albeit with a small number of patients [8]. Another recent British prospective study on a large cohort of patients (*n* = 299) assessed the safety of RFID tags, but made no comparison with WL [9]. Two on-going prospective, controlled studies assessing RFID localization are currently underway, but their results are not yet available [10, 11]. All these studies, most included in a recent meta-analysis, have concluded that RFID tags are a potential alternative to hook wire and should be considered for localizing non-palpable breast lesions [12].

Patient comfort is a crucial factor to consider when managing oncology patients, and new technologies like RFID tags have the potential to improve it. According to the literature, this is the first trial to report on patient satisfaction with RFID localization, compared to the pre-existing standard technique of WL. One RFID localization system appears as an interesting alternative to WL: the LOCalizer™ device (with CE and Food & Drug Administration accreditation). It is based on a radio-opaque 11 × 2 mm tag carried by a 12-gauge needle that can be positioned under ultrasound or stereotactic guidance. The tag can be detected up to 6 cm away whereby it re-emits a radiofrequency signal when excited at a wavelength of 134 kHz. For surgeons the handheld reader displays the distance to the tag in millimetres. Each tag can be placed in the breast at any time prior to or on the day of surgery. This device aims to improve patient comfort, enhance surgical convenience, and reduce the risk of migration. Additionally, radiologists are not dependent on the surgical schedule. The oblong shape of the tag should reduce the risk of accidental blood exposure, and damage to equipment.

Therefore, our study set out to evaluate patient satisfaction in the setting of preoperative non-palpable breast lesion localization using RFID tags (LOCalizer™ device, Hologic, Santa Carla, CA, USA).

## Methods

### Study design and objectives

The RFID trial was a single-centre, non-interventional, non-randomized, controlled, prospective observational study. The aim of the study was to ascertain the superiority of the RFID technique in terms of patient satisfaction over the gold standard technique. It also aimed to determine surgeons’ and radiologists’ perceptions of the RFID technique compared to the gold standard. We have previously published the entire detailed protocol [13].

### Study population

Eligible patients were adults (> 18 years old) requiring conservative breast surgery for a histologically-proven, non-palpable breast lesion (including cancer and B3 lesions [14]), with an indication for pre-operative localization, and they were to be able to provide informed consent, and affiliated to the French social security system. The non-inclusion criteria were the presence of multiple breast lesions or multiple localizations, pregnancy, inability to provide informed consent, or refusal to participate. Participants had the option to withdraw from the trial at any time.

Patient enrolment and the study processes are detailed in the study protocol [13]. In brief, patients completed the Hospital Anxiety and Depression Scale (HADS) questionnaire at inclusion to detect any anxiety or depressive disorders [15]. They also completed a Visual Analogue Scale (VAS) at two time points: immediately after the placement of the device (RFID tag or wire) and at the time of the postoperative consultation 1 month after surgery. Radiologists and surgeons filled in dedicated questionnaires after the pre-operative localization and after surgery. Patients were followed from the time of inclusion to one month after surgery.

**Fig. 1:**
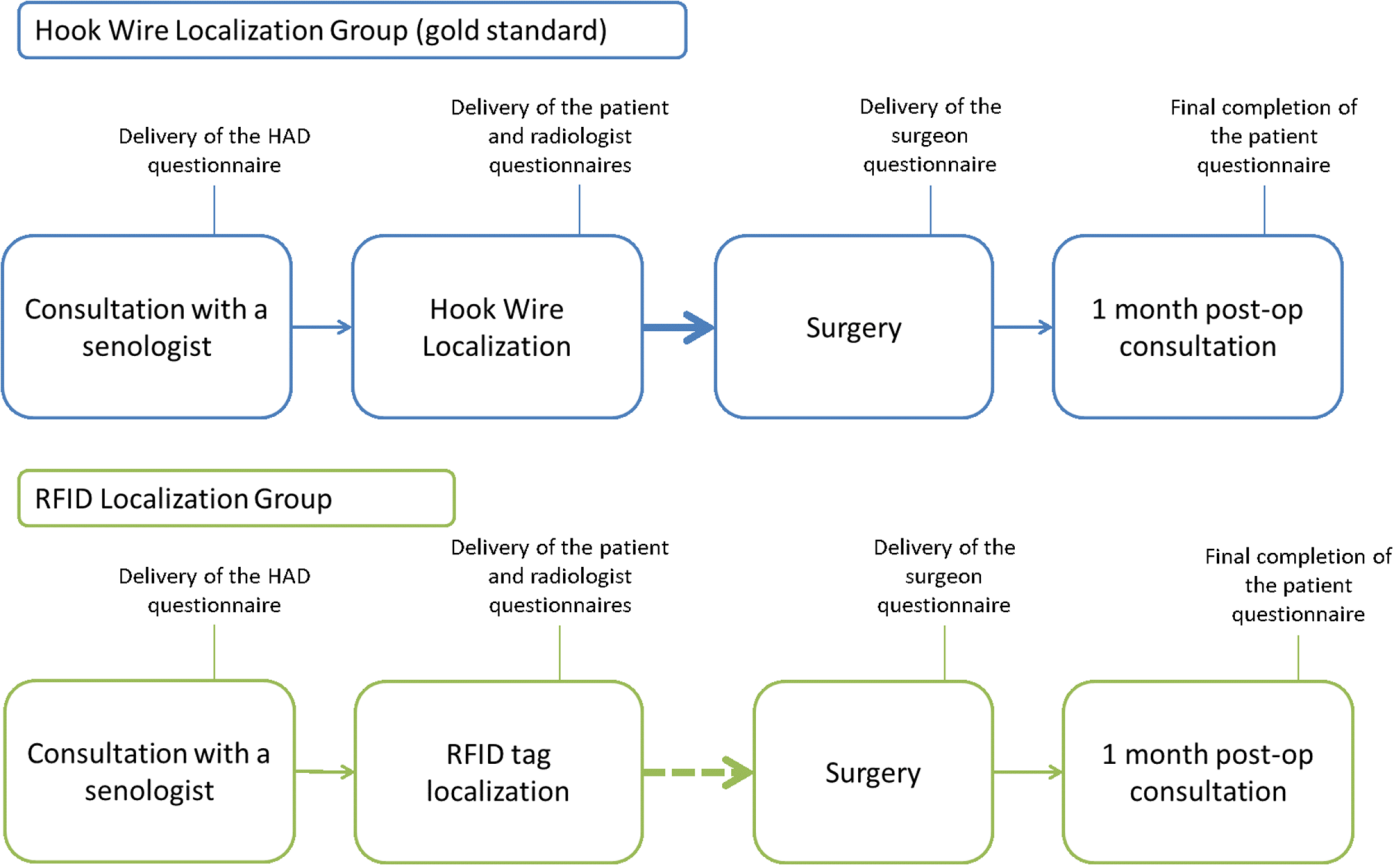
Study design. Patients completed the HADS questionnaire at inclusion during their senology consultation. They also completed meaures on a visual analogue scale at two time points: immediately after the placement of the device (RFID tag or wire) and during the postoperative consultation 1 month after surgery. Radiologists and surgeons also filled in dedicated questionnaires, after the pre-operative localization and after surgery. The patients were followed from the time of inclusion to one month after surgery

The study design is presented in Fig. 1. The preoperative location procedure requires close coordination between the radiology and surgery teams. So, we opted for sequential accrual over time, starting with 20 patients in the wire group, followed by 20 patients in the RFID group before repeating this pattern. A total of 80 inclusions were planned. We estimated the duration of patient enrolment at about 26 months. The RFID tags were placed during the senology consultation at least one week before surgery, while the wires were placed the day before or on the day of the surgery.

### Localization procedures

The localization procedures were performed using either the LOCalizer™ wire-free guidance RFID system (designed by Hologic©) for the RFID group or the repositionable hook wire Duo System (designed by SOMATEX^®^) for the wire group. These procedures were conducted under ultrasound or stereotactic guidance, according to the visibility of the lesion, using respectively a Toshiba Aplio i600 or a Selenia^®^ Dimensions Mammography System. The ultrasonographic probes used ranged from 7 MHz to 14 MHz. Lidocaine 1% local analgesia was administered systematically for all the patients. Under stereotactic guidance, breast compression ranged from 50 to 70 N without exceeding 200 N. To place the RFID tag, a small incision in the skin was required, while this step was not necessary for wire placement. Immediately after placement, two orthogonal mammograms were performed to ensure correct localization.

A migration of the device was defined as a distance > 5 mm from the target lesion at the time of device deployment. If a wire was replaced because it was more than 5 mm away from the lesion, it was considered as a case of device migration. Positive margins were defined as the tumour in contact with the edge of the surgical specimen for invasive cancer (also known as “ink on tumour”), or the tumour located less than 2 mm from the edge of the surgical specimen for ductal carcinoma in situ. If the circumferential or selective shave margins were used, the margin status was assessed on the basis of these shave margins.

### Questionnaires

**Fig. 2:**
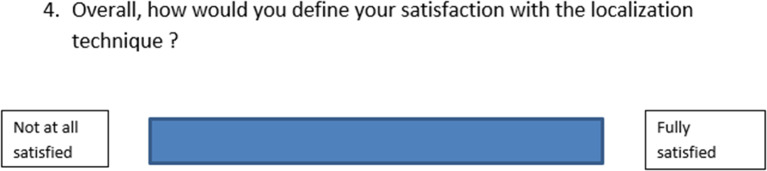
Visual analog scale to assess satisfaction

The evaluation of our variables of interest was based on the analysis of several questionnaires: the HADS and the visual analogue scales that were developed as part of this study in order to collect the clinicians’ opinions and the patients’ feedback, partly based on validated models like the VAS for pain. The VAS used to assess patient satisfaction is shown in Fig. 2. Patient scores were converted into a continuous score ranging from 0 to 10 points by measuring the distance from the left edge. Using a cross product, the scores were plotted out of 10. All these tools are available in the appendix.

### Objectives and outcomes

The main objective of the RFID trial was to ascertain the superiority of the RFID localization technique in terms of patient satisfaction, over WL. The primary outcome was patient satisfaction with the preoperative localization, evaluated using a VAS. The secondary objectives and outcomes are detailed in Table 1.

**Table 1:** Secondary objectives and outcomes

Objectives	Outcomes	Source Data
To assess the patients’ pain and stress according to the device used	The patients’ pain and stress scores in relation to the preoperative identification technique, assessed using a visual analogue scale.	- Patient questionnaire completed after the placement - Patient questionnaire completed during the postoperative consultation
To compare the radiologists’ perception of the localization by RFID to the gold-standard	The sonographic visualization of the needle and the device with ultrasound guidance and the ease of insertion of the device, determined by the radiologist	- Radiologist questionnaire
To compare the surgeons’ perceptions of localization by RFID to the gold-standard	The surgeons’ scores for precision and operative comfort associated with the device	- Surgeon questionnaire
To demonstrate the non-inferiority of the RFID technique over the gold standard in terms of performance	The occurrence or non-occurrence of device migration, a displacement during surgery, histological data with the presence of the lesion in the surgical specimen, invasion of the margins and the resulting rate of revision surgery	- Surgeon questionnaire - Anatomo-pathological report on the surgical specimen

### Statistical analysis

The sample size was calculated based on a minimal important difference of 20% and at least a 1-point difference in the satisfaction score. We determined that 30 patients per group would make it possible to show a minimum effect size of 0.75, for an estimated standard deviation of about 1.5 points, with 80% power, and a type-I error at 5%. To compensate for potential non-evaluable patients and missing data, we finally decided to include 40 patients in each group, resulting in a total of 80 patients.

The main analysis involved comparing the patient satisfaction scores between the two groups, RFID vs. wire, using the Wilcoxon-Mann-Whitney test. Before the study began, we pre-specified anxiety as a possible confounder. There was no difference in anxiety between the two groups, as measured by the HADS at inclusion – mean (SD) HADS anxiety score of 8 (4.3) in the WL group and 7.9 (4.7) in the RFID group (*p* = 0.92, Welch’s t-test), hence no matching was required for the comparative analysis.

The secondary analysis involved comparing the scores from the patient, surgeon and radiologist measures between the two study groups, using either Wilcoxon-Mann-Whitney’s test or Fisher’s exact test, with multiple testing corrections (Holm) on the patient questionnaire items (principal outcome). For the Wilcoxon-Mann-Whitney test, we reported effect size as the probabilistic index (PI, also called Mann-Whitney parameter, defined as the probability that the outcome of a subject with RFID will exceed the outcome of a subject undergoing WL), with compatible 95% CIs using the *asht* R package [16].

## Results

### Recruitment and follow-up

**Fig. 3:**
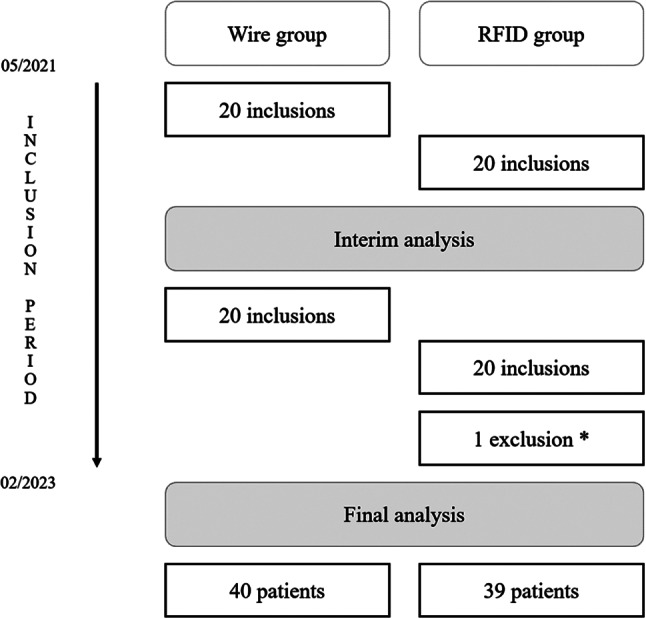
Flow chart. * One patient underwent a substantial migration during RFID tag deployment, requiring the placement of a wire to mark the correct site

From May 2021 to February 2023, 80 patients were sequentially enrolled in Jean PERRIN comprehensive cancer centre. Initially, 20 patients were included in the wire group, followed by 20 patients in the RFID group. An interim analysis showed that there was no need to readjust the number of subjects required. The same pattern was then repeated for the subsequent inclusions. A flow chart of the procedure is presented in Fig. 3. A substantial migration of about 15 mm of the RFID tag was observed in one patient during deployment, requiring the placement of a wire to mark the correct site. This patient was thus excluded from the analysis. In all, 80 patients were included, 40 in each group, and 79 patients were assessed, 40 in the wire group and 39 in the RFID group.

### Patient characteristics

The baseline clinical and pathological characteristics of the population and their non-palpable breast lesions are presented in Table 2. All patients were women. The two groups were similar in terms of age, HADS score, mammographic and sonographic size of the lesion and histology of the lesion derived from a needle-assisted biopsy. The study population was 60 years old on average. Although B3 lesions were accepted, 77% of the patients presented a malignant lesion with a T1C classification (10–20 mm). No anxiety or depressive disorders were found at inclusion. Ultrasound was the most widely used guidance technique in both RFID and WL groups, for 36 (92%) and 31 (77.5%) patients (*p* = 0.11) respectively, as shown in Table 3.

**Table 2:** Patient characteristics

Item		Total	WL		RFID
Age (yr), — mean ± SD		*61 ± 12* *n* = 79	*60 ± 12* *n* = 40		*61 ± 12* *n* = 39
HADS score		*n* = 79	*n* = 40		*n* = 39
	Anxiety score, mean ± SD	7.95 ± 4.5		8 ± 4.3	7.9 ± 4.7
	Depression score, median (IQR)	2 (3.5)		2 (3)	3 (5)
	Total HADS score, mean ± SD	11.2 ± 6.7		10.9 ± 5.7	11.6 ± 7.7
Side of the lesion, no. (%)		*n* = 79	*n* = 40		*n* = 39
	Right	39 (49)		18 (45)	21 (54)
	Left	40 (51)		22 (55)	18 (46)
Malignancy on diagnostic biopsy, no. (%)		*n* = 79	*n* = 40		*n* = 39
	B3 lesion	18 (23)		6 (15)	12 (31)
	Malignant	61 (77)		34 (85)	27 (69)
Histological type on biopsy, no. (%)		*n* = 70		*n* = 37	*n* = 33
	DCIS	19 (24)		10 (25)	9 (23)
	IDC	44 (56)		22 (55)	22 (56)
	ILC	7 (9)		5 (13)	2 (5)
Size (mm) of the lesion on mammogram, median (IQR)		12 (6.5) *n* = 55		15 (9) *n* = 23	12 (5.3) *n* = 32
Size (mm) of the lesion on sonogram, median (IQR)		11 (8) *n* = 62		13 (11) *n* = 31	11 (6) *n* = 31

**Table 3:** Radiologists’ questionnaire

Item		Total	WL	RFID	*p*-value
Localization mode, no. (%)		*n* = 79	*n* = 40	*n* = 39	0.11†
	Ultrasound guidance	67 (85)	31 (77.5)	36 (92)	
	Stereotactic guidance	12 (15)	9 (22.5)	3 (8)	
Needle US visibility, no. (%)		*n* = 68	*n* = 31	*n* = 37	0.02†
	Very poor	1 (2)	0	1 (3)	
	Average	5 (7)	5 (16)	0	
	Good	30 (44)	15 (48)	15 (40)	
	Excellent	32 (47)	11 (36)	21 (57)	
Device US visibility, no. (%)		*n* = 68	*n* = 31	*n* = 37	0.05†
	Very poor	1 (1.5)	0	1 (3)	
	Poor	1 (1.5)	1 (3)	0	
	Average	11 (16)	7 (23)	4 (11)	
	Good	27 (40)	15 (48)	12 (32)	
	Excellent	28 (41)	8 (26)	20 (54)	
Ease of placement, no. (%)		*n* = 79	*n* = 40	*n* = 39	0.12†
	Difficult	2 (2.5)	0	2 (5)	
	Intermediate	13 (16.5)	5 (12.5)	8 (21)	
	Easy	46 (58)	28 (70)	18 (46)	
	Very easy	18 (23)	7 (17.5)	11 (28)	
Migration*, no./total no. (%)		4/77 (5)	2/39 (5)	2/38 (5)	> 0.99†
Duration of the device placement, median (IQR)		15 (15)	20 (30)	15 (6)	0.01‡

* Migration is defined as the distance between device and lesion > 5 mm

† *p*-value for Fisher’s exact test comparing WL vs. RFID

‡ *p*-value for Wilcoxon-Mann-Whitney test comparing WL vs. RFID

### Patient satisfaction

**Fig. 4:**
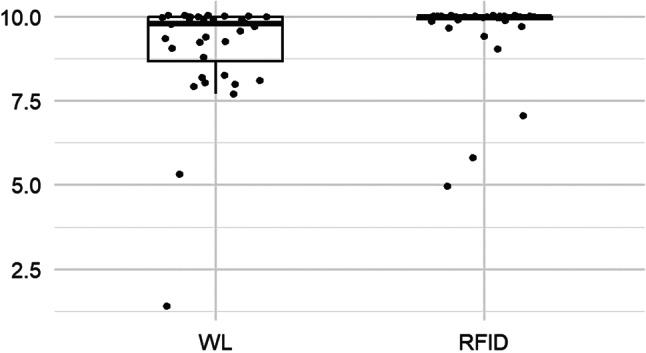
Patient satisfaction score boxplot

The patients’ satisfaction scores were globally higher with RFID localization than with the wire technique (*p* < 0.001), with the distribution of scores in the wire group showing a heavier tail towards the lower satisfaction (Fig. 4; Table 4).

**Table 4:** Patients’ questionnaires

Score	Total	WL	RFID	PI*	95% CI^§^	*p*-value†	Adj.‡ *p*-value
Pain during placement, median (IQR)	0.4 (0.95) *n* = 76	0.5 (1) *n* = 39	0.2 (0.62) *n* = 37	0.38	[0.27, 0.51]	0.07	0.14
Pain between placement and surgery, median (IQR)	0 (0.25) *n* = 76	0.1 (0.5) *n* = 39	0 (0.1) *n* = 37	0.32	[0.22, 0.44]	0.003	0.009
Stress during placement, median (IQR)	0.3 (1.2) *n* = 77	0.5 (2.4) *n* = 39	0.2 (0.67) *n* = 38	0.39	[0.28, 0.52]	0.09	0.14
Satisfaction**, median (IQR)	10 (0.6) *n* = 74	9.8 (1.3) *n* = 36	10 (0.07) *n* = 38	0.72	[0.60, 0.81]	< 0.001	0.002

* Probability index for RFID vs. WL

** Satisfaction score was recorded on the day of the post-operative consultation at 1 month after surgery

^§ 9^5% CI compatible with the probabilistic index (95% CI excludes 0.5 when *p*-value significant at 0.05 level)

† *p*-values for the Wilcoxon-Mann-Whitney test

‡ *p*-values adjusted for multiple comparisons using Holm correction

**Fig. 5:**
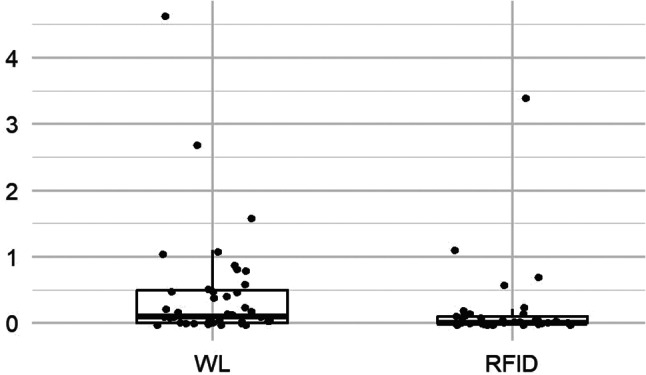
Patient pain scores between placement and surgery

**Fig. 6:**
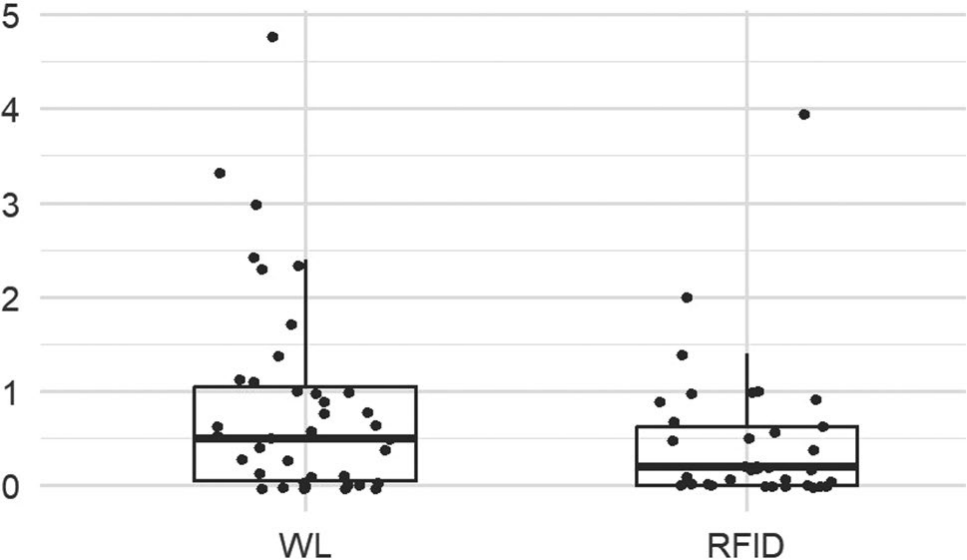
Patient pain scores during placement

**Fig. 7:**
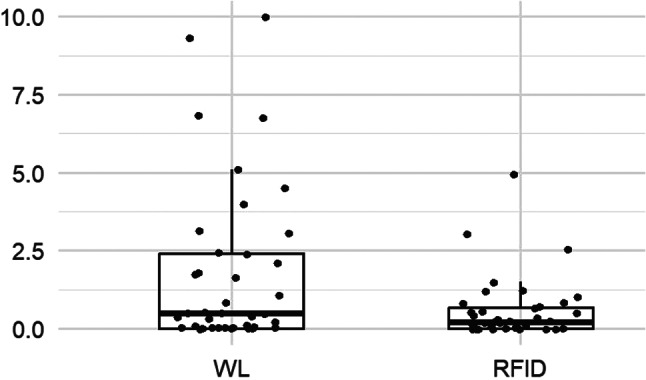
Patient stress scores during placement: boxplot

In the RFID group, as shown in Fig. 5, the pain score between device placement and surgery was lower than in the wire group, but with a median difference of only 1% (*p* = 0.009). No statistically significant difference was observed for pain during placement (*p* = 0.07) (Fig. 6) or for stress generated by the procedure (*p* = 0.09) (Fig. 7). All results from the patient questionnaires are shown in Table 4.

### Clinician satisfaction

No significant difference was found in terms of ease of placement for radiologists, as shown in Table 3. Under ultrasound guidance, the RFID needle was deemed to provide somewhat better visibility than WL (*p* = 0.02), and the RFID tag could be visualized slightly better (*p* = 0.05). The median difference in device placement duration was 5 min, shorter in the RFID group than in the wire group (PI = 0.34, 95% CI [0.24, 0.46], *p* = 0.01). Indeed, a WL placement took the radiologists on average 24 min against 15 min for the RFID system.

For surgeons, there was no significant difference in terms of accuracy or operative time between the two groups (*p* = 0.4, and 0.33 respectively). There was a slightly higher proportion of responses indicating “not at all comfortable” (19%) for surgical comfort in the RFID group than in the WL group (5%), although this difference was not statistically significant (*p* = 0.23) (Table 5). There was no accidental blood exposure.

**Table 5:** Surgeons’ questionnaire

Item		Total	WL	RFID	*p*-value
Accuracy, no. (%)		*n* = 77	*n* = 39	*n* = 38	0.40
	Poor	4 (5)	2 (5)	2 (5)	
	Average	6 (8)	1 (3)	5 (13)	
	Good	51 (66)	27 (69)	24 (63)	
	Excellent	16 (21)	9 (23)	7 (19)	
Surgical comfort, no. (%)		*n* = 77	*n* = 39	*n* = 38	0.23†
	Not at all comfortable	9 (12)	2 (5)	7 (19)	
	Comfortable	45 (58)	24 (62)	21 (55)	
	Very comfortable	23 (30)	13 (33)	10 (26)	
Displacement, no./total no. (%)		8/75 (11)	2/39 (5)	6/36 (17)	0.14†
Duration of the device placement, median (IQR)		40 (30) *n* = 73	40 (25) *n* = 35	45 (30) *n* = 38	0.33‡

† *p*-value for Fisher’s exact test comparing WL vs. RFID

‡ *p*-value for Wilcoxon-Mann-Whitney test comparing WL vs. RFID

### Localization efficacy

Except for one case of procedural failure during RFID tag deployment, which required a second localization device and resulted in the exclusion of one patient from the RFID group, both wire and RFID tag placements were highly accurate. In 95% of the cases, the distance from the lesion was less than 5 mm for both groups. Surgical excision was successful in all cases. There were 6 (17%) surgical displacement of the device in the RFID group compared to 2 (5%) in the wire group (OR = 3.6 (95% CI [0.6, 39.4], *p* = 0.14). No complications were reported with the wire technique or the RFID tag technique between placement and surgery.

Regarding the surgical specimens, there was no significant difference in the positive margin rate between the RFID group and the wire group (*p* > 0.99). Similarly, there was no significant difference in the occurrence of re-excision between the two groups (*p* = 0.77) (Table 6). One patient with positive margins did not undergo re-excision in the RFID group, due to a radial margin focal involvement of about 1 mm for Ductal Carcinoma In Situ (DCIS). The decision was made in a pluridisciplinary consultation meeting.

**Table 6:** Lesion characteristics on surgical specimen

Item	Total	WL	RFID		
Malignancy of surgical specimen, no. (%)	*n* = 78	*n* = 39	*n* = 39		
Non-malignant	24 (31)	12 (31)		12 (31)	
Malignant	54 (69)	27 (69)		27 (69)	
Histological type, no. (%)	*n* = 73	*n* = 36		*n* = 37	
DCIS	28 (38)	13 (36)		15 (41)	
IDC	37 (51)	18 (50)		19 (51)	
ILC	8 (11)	5 (14)		3 (8)	
Lesion volume (cm3), mean ± SD	109.6 ± 135.6 *n* = 75	125.5 ± 148.5 *n* = 40		91.5 ± 118.8 *n* = 35	
Positive margin, no. (%)	15 (19.2) *n* = 78	8 (20.5) *n* = 39		7 (17.9) *n* = 39	
Re-excision, no. (%)	14 (17.7) *n* = 79	8 (20) *n* = 40		6 (15.4) *n* = 39	

## Discussion

To our knowledge, this is the first prospective evaluation of patient satisfaction with RFID tags.

This study demonstrates a statistical difference between the WL technique and the RFID tag technique in terms of patient satisfaction. Indeed, patients who benefited from an RFID tag have presented a significantly higher satisfaction by close to the patients who received WL. Furthermore, this study shows a very good tolerance of the wire, that prevent major differences with RFID tags. Since no other study in the literature has focused on patient satisfaction with the RFID tag, we compared our results to studies evaluating other wire-free localization methods for non-palpable breast lesions versus the WL technique. Two randomized studies compared radioactive seed localization (RSL) and WL [17]; [18]. A study by Gray et al.., conducted in 2004, did not find any difference between groups (*n* = 100 in each group) whether for pain during localization (median of 2.0 on VAS scale) or for convenience of the entire process, 9.9 and 8.0 for the RSL group and the WL group (*p* = 0.09) respectively. A study by Bloomquist et al., conducted in 2016, showed that patients in the RSL group (*n* = 70) reported less pain during localization, which is consistent with our results for RFID tags, and a better global satisfaction than patients in the WL group (*n* = 55). A study by Misha et al. compared magnetic seed localization (MSL) (*n* = 128) to WL (*n* = 168). No difference between groups was shown in terms of pain during the localization. Patients undergoing MSL were significantly less anxious between localization and surgery than patients with WL (*p* = 0.009). We hypothesise that for patients undergoing WL, the sight of a thread coming out of their breast could be a source of stress. This could have a psychological impact on the perception of pain and could also affect satisfaction, unlike RFID tags, RSL or MSL, which are not actually seen by the patient. Finally, all these studies, including ours, have confirmed that WL is already well accepted by patients. Although our study showed a significantly higher satisfaction for RFID, this device does not seem to be able to provide a greater clinical benefit than what WL already provides. Improvements in WL and mammography devices over time could explain the absence of major clinical difference in satisfaction rates with the new tags. In our opinion, a mean difference of 2% in satisfaction cannot be the main factor in deciding which device to use. The choice of the device could therefore focus on other parameters, such as its efficacy and acceptance by clinicians.

It would have been interesting to create a composite score of acceptability, consisting in summing the scores from the different patient questionnaires (pain during placement, pain between placement and surgery, stress during placement and global satisfaction).

Regarding the quality of the localization, surgical displacements were more frequent in the RFID group than in the WL group, with an odds ratio of 3.6 (95% CI [0.6, 39.4], *p* = 0.14). We hypothesize that these displacements could be attributed to the non-adhesive glass surface and the oblong shape of the RFID tag despite the polypropylene cap, and to an inaccurate positioning of the tag in relation to the lesion. Eighteen per cent of the surgeons reported difficulties during surgery for patients undergoing RFID tags, and 10% reported difficulties in the WL group. This could be attributed to the perceived per-operative risk of displacement the RFID tag, and the manual reader, which detects the ends of the RFID tag rather than its centre. Singh et al.. described a similar phenomenon and reported better results when the RFID tag was placed in a deeper part of the target lesion, instead of the subcutaneous site, which may be prone to displacement during incisions and surgical exposure [19]. Twenty-three per cent of our radiologists reported difficulties in progressing with the 12-gauge RFID needle in dense breasts. In most cases involving firm masses, the tag was deployed when in contact with the lesion rather than within it. These constraints do not exist with WL. In our study, we encountered a procedural failure when deploying the RFID tag. This failure was probably due to the high calibre (12 gauge) and the blunt tip of the insertion needle, and also the shape of the tag, combined with the deep positioning of the lesion in a dense breast. Lowes et al.. previously reported three procedural failures as a result of tag migrations, which required a second deployment of the device [1]. This was also the case for Christenhusz et al., where two of their patients received a second RFID tag and four others had to finally undergo WL [20]. Margin positivity and re-excision rates have been reported for completeness, although this study did not have enough power to detect a difference for these variables. For WL, Davey et al.. reported a positive margin rate of 20.1% and a surgical re-excision rate of 17.3% in a meta-analysis involving 24 randomized controlled trials [21]. In a review by Tayeh et al.., the surgical re-excision rate for RFID localization was 13.9% [12]. In most cases, the small displacements of the device that we observed do not require a change in the patient’s management, since the tag tends to migrate to the surface of the lesion. During a tumorectomy, the surgeon removes the marker until the deep plane, which allows him to remove the lesion even if the marker has migrated to the surface. For this reason, we did not observe any difference in the surgical re-excision. However, a tag moving a few millimeters upstream of the lesion can decrease radiologists’ confidence in the device, Practitioners should therefore be aware of the migration risk associated with this device when using it.

We found the same difference in terms of placement times for the two devices as in the literature. Indeed, placing the RFID tag required an average of 10 min less for radiologists than for WL. Christenhusz et al.. were able to demonstrate an even shorter RFID tag deployment time with an average of about 5 min, versus 14 min for the RFID group in our study [20]. This discrepancy with the literature could be explained by the way we timed the placement of the tag, which included the time of installation of patient and sterile equipment. This saving of medical time is a real advantage, not only logistically but also financially, despite the more onerous cost of the tag compared to wire. For some vulnerable patients who cannot go back home after WL and who require hospitalization the night before surgery, receiving the tag several days before their operation makes surgery possible in ambulatory care. A large medical and economic study taking all these parameters into account is warranted to evaluate these aspects.

The LOCalizer™ technology could be further optimized in its design to improve clinical performance. We support a suggestion by Tayeh and his collaborators [12], to reduce the gauge of the introduction needle to a 16 gauge to facilitate deployment in dense tissues, and to remove the glass envelope of the RFID, if this is technically possible, because it can contribute to its migratory potential [12]. In addition to the shape and calibre of the Localizer™ tag and needle, RFID localization has other limitations. In particular, it should be avoided among patients with pacemakers and defibrillators because of the risk of electromagnetic interference [22, 23] and among patients with very deep lesions, because of the current theoretical maximum detection range of 6 cm. It also generates a magnetic vacuum artefact, which could limit the interpretation of Magnetic Resonance Imaging (MRI) [10].

Patient comfort is at the heart of cancer care today. This is the first study to evaluate patient comfort when using RFID tags. It is also the largest prospective controlled study to date comparing two localization devices: RFID and WL. The main strengths of our study are that, unlike other studies on satisfaction, our data from patient, surgeon and radiologist questionnaires was almost complete, ranging from 92 to 100%, giving us sufficient power to demonstrate a significant superiority of RFID tags in terms of patient comfort. A study with an even larger cohort could have demonstrated the same clinical difference. Among the other limitations, the observational design of the study in a single centre and the non-randomized controlled trial can be criticized, although we tried to mitigate any selection bias by sequential accrual. Unfortunately, we were unable to validate the use of VAS in another study to measure the patient satisfaction, which is a limitation of our study. Moreover, due to its non-randomized, observational design, we could have added other potential confounders, in addition to anxiety, to adjust the assessment of patient satisfaction. Our results need to be weighed up against the limited experience of RFID localization and our clinicians’ increasing learning curve. Nevertheless, for radiologists, the learning curve is short because the insertion process is the same as for many other lesion markers used in breast radiology. It would also be interesting to envisage multiple localizations, which sometimes occur in clinical practice.

## Conclusion

RFID tags are well accepted by patients and clinicians. RFID tags are a reliable alternative to wires, while simplifying the organization of the patients’ healthcare trajectories and reducing the radiologists’ medical time. The miniaturization of the RFID tag and the introduction of the needle seem to us to be an improvement, as they could enable certain limitations to be overcome. It would be interesting to conduct a medical-economic study to determine which device is the cheaper.

## Electronic supplementary material

Below is the link to the electronic supplementary material.

Supplementary Material 1

Supplementary Material 2

Supplementary Material 3

## Data Availability

The datasets used and/or analysed in the current study are available from the corresponding author on reasonable request.

## Abbreviations

BRCA: Breast cancer gene
CI: Confidence interval
DCIS: Ductal carcinoma in situ
HADS: Hospital anxiety and depression scale
IDC: Invasive ductal carcinoma
ILC: Invasive lobular carcinoma
IQR: Interquartile range
MRI: Magnetic resonance imaging
MSL: Magnetic seed localization
No: Number
OR: Odds ratio
RFID: Radiofrequency identification
SBR: Scarff-bloom-Richardson
SD: Standard deviation
US: UltraSound
VAS: Visual analogue scale
WL: Wire localization
Yr: Year
